# Palmitic acid–rich oils with and without interesterification lower postprandial lipemia and increase atherogenic lipoproteins compared with a MUFA-rich oil: A randomized controlled trial

**DOI:** 10.1093/ajcn/nqaa413

**Published:** 2021-03-01

**Authors:** Charlotte E Mills, Scott V Harding, Mariam Bapir, Giuseppina Mandalari, Louise J Salt, Robert Gray, Barbara A Fielding, Peter J Wilde, Wendy L Hall, Sarah E Berry

**Affiliations:** Department of Nutritional Sciences, School of Life Course Sciences, Faculty of Life Sciences & Medicine, King's College London, London, UK; Department of Food and Nutritional Sciences, University of Reading, Reading, UK; Department of Biochemistry, Memorial University of Newfoundland, St. John's, Newfoundland and Labrador, Canada; Department of Nutritional Sciences, School of Life Course Sciences, Faculty of Life Sciences & Medicine, King's College London, London, UK; Food Innovation and Health Programme, Quadram Institute Bioscience, Norwich, UK; Department of Chemical, Biological, Pharmaceutical, and Environmental Science, University of Messina, Messina, Italy; Food Innovation and Health Programme, Quadram Institute Bioscience, Norwich, UK; Department of Nutritional Sciences, School of Life Course Sciences, Faculty of Life Sciences & Medicine, King's College London, London, UK; Department of Nutritional Sciences, University of Surrey, Surrey, UK; Food Innovation and Health Programme, Quadram Institute Bioscience, Norwich, UK; Department of Nutritional Sciences, School of Life Course Sciences, Faculty of Life Sciences & Medicine, King's College London, London, UK; Department of Nutritional Sciences, School of Life Course Sciences, Faculty of Life Sciences & Medicine, King's College London, London, UK

**Keywords:** lipid, interesterification, palmitic acid, rapeseed oil, metabolism, healthy adults, postprandial lipemia

## Abstract

**Background:**

Interesterified (IE) fats are widely used in place of *trans* fats; however, little is known about their metabolism.

**Objectives:**

To test the impact of a commonly consumed IE compared with a non-IE equivalent fat on in vivo postprandial and in vitro lipid metabolism, compared with a reference oil [rapeseed oil (RO)].

**Methods:**

A double-blinded, 3-phase crossover, randomized controlled trial was performed in healthy adults (*n* = 20) aged 45–75 y. Postprandial plasma triacylglycerol and lipoprotein responses (including stable isotope tracing) to a test meal (50 g fat) were evaluated over 8 h. The test fats were IE 80:20 palm stearin/palm kernel fat, an identical non-IE fat, and RO (control). In vitro, mechanisms of digestion were explored using a dynamic gastric model (DGM).

**Results:**

Plasma triacylglycerol 8-h incremental area under the curves were lower following non-IE compared with RO [–1.7 mmol/L⋅h (95% CI: –3.3, –0.0)], but there were no differences between IE and RO or IE and non-IE. LDL particles were smaller following IE and non-IE compared with RO (*P* = 0.005). Extra extra large, extra large, and large VLDL particle concentrations were higher following IE and non-IE compared with RO at 6–8 h (*P* < 0.05). No differences in the appearance of [^13^C]palmitic acid in plasma triacylglycerol were observed between IE and non-IE fats. DGM revealed differences in phase separation of the IE and non-IE meals and delayed release of SFAs compared with RO.

**Conclusions:**

Interesterification did not modify fat digestion, postprandial lipemia, or lipid metabolism measured by stable isotope and DGM analysis. Despite the lower lipemia following the SFA-rich fats, increased proatherogenic large triacylglycerol-rich lipoprotein remnant and small LDL particles following the SFA-rich fats relative to RO adds a new postprandial dimension to the mechanistic evidence linking SFAs to cardiovascular disease risk.

## Introduction

Interesterification is a process extensively used by the food industry to produce solid fats with suitable functionality for diverse applications, including bakery, spreads, and confectionery products. Interesterification involves the chemical or enzymatic redistribution of fatty acids across the *sn*-1, *sn*-2, and *sn*-3 positions on the glycerol backbones of triacylglycerols (TAG) (1). This process is typically used in conjunction with fractionation and full hydrogenation to form a *trans-*free “hard stock.” The resulting hard stock is blended in different proportions with vegetable oil [typically rapeseed oil (RO)] to produce fats with desirable functional properties. Subsequently, interesterified (IE) fats *1*) are alternatives to harmful *trans* fatty acid–containing partially hydrogenated fats, *2*) are lower in saturated fat compared with traditional fats (e.g., butter), and *3*) give the food industry greater control over the physical and organoleptic properties of the fat (1). Despite their widespread use, little is known about how IE fats are metabolized by the body and their subsequent health effects, with most previous work focusing on IE fats, which are not commonly used commercially [previously discussed (1)].

Many of the chronic health effects of dietary fats are underpinned by acutely changing lipid-induced circulating metabolites. Following fat consumption, there is an acute elevation in circulating TAG [an independent risk factor for cardiovascular disease (CVD)] (2–4), which remain elevated for ∼8 h. Alongside this, there is a perturbation in circulating atherogenic lipoproteins (5) and increases in circulating inflammatory, oxidative stress, and hemostatic measures, affecting endothelial function (6) and CVD risk (7). Given that we consume multiple meals throughout the day, we spend the majority of our day in a postprandial state. Therefore, determining an individual's postprandial response to different dietary fats is a powerful tool for understanding their chronic health effects.

We (8–12) and others (13, 14) have previously shown that interesterification influences postprandial fat handling and lipemic responses, reviewed elsewhere (15, 16). However, because interesterification simultaneously changes the solid fat content and increases the proportion of SFAs in the *sn*-2 position, it is unclear whether observed differences in postprandial lipemia are due to changes in physical properties (solid fat profile) or positional composition (*sn*- positional composition), which may affect rates of digestion and metabolism (15).

Therefore, the purpose of this study was to *1*) investigate the impact of a commonly consumed IE fat compared with non-IE fat and a reference high-MUFA oil on postprandial lipid metabolism in a randomized controlled trial (in vivo) and *2*) use a dynamic gastric model (DGM) of digestion to identify potential mechanisms underpinning in vivo observations (in vitro). We hypothesized that interesterification would increase postprandial lipemia over 8 h possibly due to alterations in the physical properties of the fat.

## Methods

### Participants

Ethical approval for the Inter-Met study was obtained from King's College London Research Ethics Committee (HR-16/17-4397), and written informed consent was provided by participants. The study was registered at clinicaltrials.gov as NCT03191513 and conducted in accordance with the ethical standards laid down in the 1964 Declaration of Helsinki and its later amendments. Healthy adult men and women (aged 45–75 y) were recruited via advertisements at King's College London and the surrounding area between June and November 2017. Interested participants were sent an initial screening questionnaire, and if deemed eligible from this, they were invited for a screening visit at the Metabolic Research Unit (MRU) at the Department of Nutritional Sciences, King's College London, following an overnight fast (12 h). Their weight, height, waist and hip circumference, percentage body fat, seated blood pressure, glucose, insulin, liver function, and lipid profile were measured as outlined previously (11). Participants were requested to complete a 3-d diet diary to assess their habitual nutritional intakes (2 weekdays and 1 weekend day) using dietary assessment software (Nutritics Ltd). Exclusion criteria were as follows: medical history of myocardial infarction, angina, thrombosis, stroke, cancer, liver or bowel disease or diabetes, BMI (in kg/m^2^) <20 or >35, fasting plasma cholesterol ≥7.5 mmol/L, TAG >3 mmol/L or glucose >7 mmol/L, blood pressure ≥140/90 mmHg, current use of antihypertensive or lipid-lowering medications, alcohol intake exceeding a moderate intake (>224 g/wk), and current cigarette smoker. The study was powered for 20 people (10 men and 10 women separately) to complete, to detect a mean difference in plasma TAG incremental area under the curve (iAUC) of 40 mmol/L/min with an SD of differences of 32 mmol/L/min, at 95%, *P* < 0.05, and a mean difference of 0.30 mmol/L in peak concentrations with an SD of differences of 0.33 mmol/L, at 80%, *P* < 0.05, using previous data from our group (12).

### Randomized controlled trial design and test fats

A randomized, double-blind, 3-phase crossover design was used to compare test meals differing in the type of fat, with a minimum 1-wk washout period. Each test meal consisted of a muffin and a milkshake containing 3.75 MJ (896.5 kcal), 16 g protein (7% energy), 88 g carbohydrate (39% energy), and 50 g test fat (54% energy); all meals were similar in appearance and taste. A subgroup (*n* = 9) of the participants received muffins with 75 mg [1,1,1]-^13^C glyceryl tripalmitate (Cambridge Isotope Laboratories); these participants had a minimum 4-wk washout between visits.

#### Outcome variables

The primary outcome was iAUC_0–8_ for TAG with other TAG parameters as secondary outcomes. Other secondary outcomes included plasma TAG fraction fatty acid composition and nonesterified fatty acids (NEFAs), chylomicron TAG concentration apolipoprotein B48 (apoB48), ^13^C TAG, breath CO_2_^13^C, lipoprotein particle size, and number.

The test fats were non-IE palm stearin (PSt)/palm kernel (PK), IE PSt/PK (blended at a ratio of 80:20 PSt/PK, made from the same batch of oil), and RO (control) (all provided by ADM Speciality Oils & Fats Ltd). Both the non-IE and the IE fat blends had a similar fatty acid composition, but the proportions of palmitic acid in the *sn*-2 position were 33 mol% and 53 mol%, respectively (**Table 1**). Measurement of the solid fat content by NMR (European laboratories of ADM Hamburg AG) of the non-IE and IE gave values shown in Table 1; notably (close to body temperature), they were 25.8% and 22.7% at 35°C and 19.5% and 11.5% at 40°C, respectively. Participants consumed the test meals in a random order determined by computer randomization allocated sequentially by a technician independent of the study; treatment allocation was blinded from the study participants and investigators. On the day preceding each test meal, participants were instructed not to participate in strenuous exercise and to avoid caffeine, alcohol, and foods high in fat. They were provided with a standardized low-fat meal (containing <20 g fat) to consume as their evening meal before 22:00. Participants attended the MRU between 08:00 and 10:00 the following morning. A cannula was inserted into the forearm (antecubital vein), baseline samples were taken, and the test meal was consumed within 10 min. Additional venous blood samples were collected at hourly intervals until 8 h after meal consumption. Participants had access to water to drink at regular intervals throughout the study day. Following the 3-h blood sample, participants consumed a fat-free lunch meal of 1.7 MJ consisting of 190 g fat-free yogurt and a banana, as used in our previous studies to make the procedure tolerable (11).

**TABLE 1:** Fatty acid composition of the experimental fats (wt%)^1^

Fatty acid	Non-IE^2^	IE^2^	Rapeseed oil
12:0	8.7	8.5	0.0
14:0	3.9	3.8	0.0
16:0	49.6	48.8	6.8
18:0	4.5	4.5	3.0
18:1n–9 *cis*	25.5	26.6	62.0
18:2n–6 *cis*	5.3	5.4	18.6
18:3n–3 *cis*	0.1	0.1	9.7
Solid fat content at 10°C, %	74.0	79.3	0.0
Solid fat content at 20°C, %	54.6	59.7	0.0
Solid fat content at 25°C, %	44.2	48.4	0.0
Solid fat content at 30°C, %	34.0	35.3	0.0
Solid fat content at 35°C, %	25.8	22.7	0.0
Solid fat content at 40°C, %	19.5	11.5	0.0

1 Data are presented as percent base on total weight.

2 An 80:20 (%) blend of palm stearin/palm kernel.

### Analytical methods

#### Test fat analysis

The fatty acid composition of the test fats and positional composition were determined as previously described (8). The composition of the test fats is shown in Table 1.

#### Circulating lipid analysis

TAG, NEFA, and fatty acid composition was performed on blood samples taken hourly between 0 and 8 h. NEFA (WAKO NEFA-HR) and TAG (Roche Diagnostics Limited) concentrations were analyzed in plasma using enzymatic assays on an ILAB-650 clinical chemistry analyzer (Instrumentation Laboratories), with interassay CVs of 2.3% and 2.4%, respectively. Plasma TAG fraction fatty acids were analyzed by gas–liquid chromatography, as previously described (17). Total lipoprotein profiles (size and particle number) were determined at 0, 2, 4, 5, 6, and 8 h by NMR performed on serum by Nightingale Health (18), using the 2016 panel. Chylomicrons were separated from plasma at 2, 4, and 6 h after baseline by ultracentrifugation. ApoB48 analysis was conducted by immunoassay (Affinity Biomarker Labs), representing apoB48 concentrations in these chylomicron fractions. Lipids were also extracted from the chylomicrons and the TAG fraction isolated by thin-layer chromatography (TLC). *Sn-2* fatty acid composition of the test fat was analyzed by gas chromatography on fatty acid methyl esters (FAMEs) as outlined previously (8).

Samples for all analyses were collected into EDTA tubes, other than for lipoprotein profiles, which were collected in serum separating tubes. Blood was centrifuged, and resulting plasma or serum was stored at –80°C for later analysis.

### Stable isotope analysis

TAG fractions were isolated from plasma by TLC, and TAG FAME concentrations were analyzed by gas chromatography–isotope ratio mass spectrometry (GC-IRMS) to determine ^13^C enrichment over the postprandial period. Plasma fatty acid and breath samples were analyzed for ^13^C/^12^C ratio by IRMS (Delta V Plus; Thermo Scientific) and expressed as δ^13^C in units of ‰ or per mil, calibrated using a FAME fatty acid standard (Indiana University, Bloomington) that had been normalized to Vienna Pee Dee Belemnite (VPDB). The isotopic enrichment of plasma and breath samples was expressed as percent dose recovered (PDR) for plasma (PDR_Plasma_) and breath CO_2_ (PDR_Breath_) (18). Briefly, the ^13^C/^12^C ratio of both the plasma and breath samples was expressed as the delta (δ^13^C; in units of ‰ or per mil) compared with the international standard VPDB, with a ^13^C/^12^C ratio of 0.0112372. The ^13^C enrichment in the plasma and breath samples was calculated as

(1) $$\begin{eqnarray*}
{{\rm{\delta }}^{{\rm{13}}}}{\rm{C}}\,{\rm{ = }}\,\frac{{{{\rm{R}}_{\rm{S}}} - {{\rm{R}}_{{\rm{PDB}}}}}}{{{{\rm{R}}_{{\rm{PDB}}}}}} \times {\rm{ }}1000,
\end{eqnarray*}$$

where R_S_ is the ^13^C/^12^C ratio of the sample, and R_PDB_ is the ^13^C/^12^C ratio of VPDB.

Using the δ^13^C, we then calculated the atom percent excess of the tracer and further calculated the PDR of the tracer from β-oxidation (19, 20). Briefly, PDR_Breath_ was calculated using the following formula:

(2) $$\begin{eqnarray*}
{\rm{PD}}{{\rm{R}}_{{\rm{Breath}}}}{\rm{ = }}\frac{{\begin{array}{*{20}{c}} {{\rm{APE }}\left( {{\rm{atom\,\, percent\,\, excess}}} \right){\rm{ }} \times {\rm{ VC}}{{\rm{O}}_{\rm{2}}}} \end{array}}}{{{\rm{mmo}}{{\rm{l}\,\,}^{{\rm{13}}}}{\rm{C\,\, administered}}}} \times 1000,
\end{eqnarray*}$$

where VCO_2_ was calculated by multiplying the CO_2_ production constant (300 mmol/h) by body surface area (19). The PDR_Plasma_ (^13^C recovery in plasma fatty acids) was calculated as the absolute amount of [^13^C]palmitic acid recovered in the plasma TAG pool during the postprandial period. Briefly, the following calculations were used to determine the PDR_Plasma_:

(3) $$\begin{eqnarray*}
{\rm {Dose \, (mg) \, ^{ 13}C \, recovered}} & = & {\rm {APE} \times {fatty \, acid \, pool \, size \, (mg)}} \nonumber\\ && \times \, {\rm {molecular \, weight \, of \, carbons \, }} \nonumber\\ && {\rm {in \, palmitic \, acid}}
\end{eqnarray*}$$

The fatty acid pool size was calculated according to an estimated plasma volume of 4.5% of body weight and multiplied by the fatty acid concentration and the molecular weight of the carbons present in the palmitic acid. The PDR_Plasma_ was calculated as follows:

(4) $$\begin{eqnarray*}
{\rm{PD}}{{\rm{R}}_{{\rm{Plasma}}}}\,{\rm{ = }}\frac{{\begin{array}{*{20}{c}} {{\rm{Dose\, }}{{\left( {{\rm{mg}}} \right)\,\,}^{{\rm{13}}}}{\rm{C\,\, recovered}}} \end{array}}}{{\begin{array}{*{20}{c}} {{\rm{Dose\, }}{{\left( {{\rm{mg}}} \right)\,\,}^{{\rm{13}}}}{\rm{C\,\, administered}}} \end{array}}} \times 100\% ,
\end{eqnarray*}$$

where the dose ^13^C recovered was derived from the previous equation, and the dose ^13^C administered was the proportion of the 75 mg [1,1,1]-^13^C glyceryl tripalmitin, which was [^13^C]palmitic acid.

### Simulated gastric digestion

The mechanisms of gastric emptying of IE/non-IE fats from the stomach were investigated in vitro. Non-IE fat, IE fat, and RO test meals used in the in vivo study were replicated and tested using a simulated oral phase (21) and gastric conditions by DGM, which is a fully dynamic, physical, and biochemical simulation of the human stomach (22). The DGM incorporates the inhomogeneous gastric mixing, antral shearing, and rate of delivery to the duodenum, with addition of gastric acid and enzymes in a normal physiologic range and flow rates modified in real time. Based on eco planar MRI and human studies data on nasogastric and nasoduodenal aspiration (23–26), the DGM provides a realistic tool for the simulation of human gastric digestion. For the oral phase, in duplicate, 150-g muffins, providing 50 g fat per test meal, were microwaved at full power for 30 s and then passed through a meat mincer (Lakeland) to simulate mechanical oral breakdown, producing chewed particles ≤3.0 mm. Simulated salivary fluid (SSF; 75 mL) at pH 6.9 (0.15 M NaCl, 3 mM urea) and human salivary amylase (2700 U) dissolved in SSF (1 mL) were added to the minced muffins, as previously described by Mandalari et al. (21). Chewed muffins were mixed with a homemade milkshake freshly prepared and containing skimmed milk (220 mL), strawberry milkshake powder (Nesquik; Nestlé) (15 g), and caster sugar (2 g).

The chewed materials were transferred to the DGM, where they underwent physical (massage) and biochemical processing (gradual reduction of pH and proteolysis by pepsin). For the gastric phase, a suspension of single-shelled lecithin liposomes prepared as previously described (21) was added to a gastric enzyme solution [0.15 mM NaCl, 2000 U/mL porcine pepsin, 60 U/mL gastric lipase (analogue of fungal origin; Amano Enzyme)] at a final concentration of 0.127 mM phosphatidylcholine [egg L-α-phosphatidylcholine (lecithin grade 1, 99% purity); Lipid Products]. Gastric digestions were carried out on the chewed muffin samples (520 g) in the presence of 20 mL priming acid (0.1 M HCl, 0.08 M NaCl, 0.03 mM CaCl_2_, 0.9 mM NaH_2_PO_4_). Simulated gastric acid (0.2 M HCl, 0.08 M NaCl, 0.03 mM CaCl_2_, 0.9 mM NaH_2_PO_4_) and gastric enzyme solution were mechanically secreted into the DGM throughout the digestion. Nine ∼61-g samples were emptied from the DGM at 14, 28, 42, 56, 70, 84, 98, 112, and 126 min. Gastric emptying rates were determined by the amount of fat and the fatty acid profiles of the fat emptied from the DGM over time.

### Statistics

Statistical analysis was performed using IBM SPSS Statistics version 25.0. For each of the analyses described below, the assumptions of normality and homogeneity of variance were assessed, and none of the outcomes were deemed necessary to require transformation.

The iAUC was calculated using the trapezoidal rule. The iAUC, C_max_, and time point data following each treatment were analyzed using a linear mixed model. Terms in the model included treatment group, time point, period, and treatment × period as fixed effects (and treatment × time for time point analysis), participant as a random effect, and baseline values as covariates. For plasma TAG and lipoprotein particle concentrations/size, further analysis was conducted with sex and treatment × sex as fixed effects. Participants who did not complete all 3 study periods or where there were missing data at individual time points but data from at least one of the study periods or other time points in all study periods were included in the analysis by including participant as a random effect in the analysis. For the calculation of iAUC, if one value was missing over the assessment period, the AUC was generated by imputation using the average of the values available either side of the missing time point (there were 3 missing TAG data points in total). If there was >1 missing value or either of the start or end values were missing, no iAUC was calculated for that period for that participant. For all tests, the significance level was set at *P* < 0.05 (2-tailed). Post hoc analyses were made using Bonferroni adjustment for multiple comparisons.

## Results

### Participants

Twenty participants completed the study (10 men and 10 women); details of the flow of participants through the study are shown in **Supplementary Figure 1**, and baseline characteristics of the study participants are in **Table 2**.

**TABLE 2:** Baseline characteristics of trial participants^1^

Characteristic	Total (*n* = 20)	Women (*n* = 10)	Men (*n* = 10)
Age, y	58.5 ± 6.4	59.3 ± 6.4	57.7 ± 6.8
Height, m	1.72 ± 0.12	1.61 ± 0.05	1.82 ± 0.05
Weight, kg	77.2 ± 14.9	67.8 ± 12.0	86.6 ± 11.3
BMI, kg/m^2^	26.2 ± 3.8	26.0 ± 4.2	26.3 ± 3.6
Hip/waist ratio	0.88 ± 0.06	0.85 ± 0.05	0.91 ± 0.05
Systolic BP, mmHg	116.2 ± 11.3	113.6 ± 14.5	119.2 ± 5.5
Diastolic BP, mmHg	76.6 ± 9.5	72.8 ± 10.4	80.4 ± 7.2
Plasma glucose, mmol/L	5.16 ± 0.48	5.25 ± 0.52	5.06 ± 0.45
Serum total cholesterol, mmol/L	5.61 ± 0.7	5.79 ± 0.33	5.42 ± 0.92
Serum LDL cholesterol, mmol/L	3.47 ± 0.62	3.57 ± 0.31	3.36 ± 0.84
Serum HDL cholesterol, mmol/L	1.60 ± 0.33	1.62 ± 0.31	1.57 ± 3.36
Serum triglycerides, mmol/L	1.18 ± 0.47	1.31 ± 0.56	1.05 ± 0.34
Total/HDL cholesterol	3.63 ± 0.70	3.71 ± 0.73	3.55 ± 0.70
Energy MJ/d	7.8 ± 1.9	7.3 ± 1.4	8.4 ± 2.2
Protein, % energy	19.1 ± 4.0	18.6 ± 3.4	19.7 ± 4.6
Carbohydrate, % energy	47.1 ± 10.0	45.9 ± 8.2	48.5 ± 10.9
Fat, % energy	32.1 ± 8.1	34.6 ± 5.5	29.4 ± 9.6

1 Data are means ± SDs. BP, blood pressure.

### Postprandial lipemia

**FIGURE 1:**
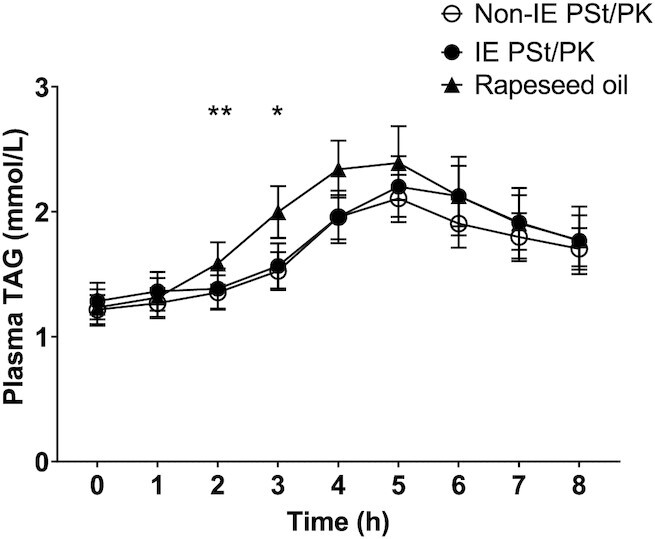
Plasma triacyglycerol (TAG) concentrations following a test meal containing 50 g interesterified (IE) palm stearin/palm kernel (PSt/PK) fat compared with a non-IE equivalent, relative to a reference rapeseed oil (RO). Data are mean (SEM), *n* = 20. Comparison of IE PSt/PK and non-IE PSt/PK by linear mixed-model analysis (dependent variable change from baseline, fixed factors of treatment, time, period, treatment × time interaction, treatment × period interaction; random effect participant; covariate baseline TAG concentrations) showed no significant treatment or treatment × time effects. Comparison of all treatments showed a significant treatment effect (*P* = 0.0008), treatment × time effect (*P* = 0.007), and treatment × period effect (*P* = 0.037). Post hoc analysis of time point differences with Bonferroni adjustment showed significant differences between both IE and non-IE fats compared with RO at 2 and 3 h (*IE compared with RO *P* = 0.004 and non-IE compared with RO *P* = 0.0002, **IE compared with RO *P* = 0.0006 and non-IE compared with RO *P* = 0.0008).

There were no differences in postprandial plasma TAG concentrations between IE and non-IE fats (**Figure 1**), measured by iAUC_(0–8 h)_, iAUC_(0–4 h)_, C_max_, and T_max_ (**Table 3**). However, in comparison with the RO (high-MUFA control oil), postprandial plasma TAG concentrations following both IE and non-IE were lower (treatment effect *P* = 0.0008 and treatment × time interaction *P* = 0.007). Relative to RO, iAUC_(0–4 h)_ was significantly lower following both IE (*P* = 0.003) and non-IE fats (*P* = 0.0003), whereas iAUC_(0–8 h)_ was significantly lower following non-IE only (*P* = 0.043). In addition, there was a trend for a higher C_max_ following RO compared with non-IE (*P* = 0.058). Analysis by sex did not reveal any significant differences in lipemic responses between men and women or treatment × sex interactions. There were no treatment effects on postprandial NEFA concentrations (**Supplementary Figure 2**).

**TABLE 3:** Postprandial incremental area under the curve (iAUC), peak concentration, and time of peak concentration for plasma triacylglycerol concentrations following an interesterified (IE) palm stearin/palm kernel (PSt/PK) fat compared with a non-IE equivalent, relative to a reference rapeseed oil (RO)^1^

Characteristic	IE PSt/PK fat	Non-IE PSt/PK fat	RO (reference)	Treatment effect (*P* value)	Mean difference IE – non-IE	Mean difference IE – RO	Mean difference non-IE – RO
iAUC_(0–8 h)_, mmol/L⋅h	3.80 (2.33, 5.27)	3.61 (3.02, 4.20)^2^	5.28 (4.08, 6.47)^2^	0.040	0.20 (–1.75, 2.14)	–1.47 (–3.74, 0.79)	–1.67^3^ (–3.29, –0.04)
iAUC_(0–4 h)_, mmol/L⋅h	0.80 (0.30, 1.30)	0.88 (0 .61, 1.16)	1.82 (1.37, 2.27)	0.0002	–0.09 (–0.72, 0.55)	–1.02^4^ (–1.73, –0.31)	–0.94^5^ (–1.43, –0.45)
Cmax, mmol/L	2.28 (1.97, 2.59)	2.26 (2.10, 2.41)	2.66 (2.31, 3.00)	0.058	0.02 (–0.36, 0.40)	–0.38 (–0.89, 0.14)	–0.40 (–0.81, 0.01)
Tmax, h	5 (4, 6)	5 (5, 5)	5 (4, 5)	0.334^6^	NA	NA	NA

1 Values are estimated marginal means with baseline values as a covariate, apart from Tmax, which are medians with limits of lower and upper quartiles, *n* = 20. Data analyzed using a linear mixed model for iAUC and Cmax (fixed factors of treatment, period, treatment × period interaction; random effect of participant; covariate baseline TAG concentrations). NA, not applicable.

2 *n* = 19 due to missing sample.

3 Mean difference is significant (*P* = 0.043).

4 Mean difference is significant (*P* = 0.003).

5 Mean difference is significant (*P* = 0.0003).

6 Pearson χ^2^ test.

### Labeled palmitic acid appearance in plasma triacylglycerol fraction and breath

**FIGURE 2:**
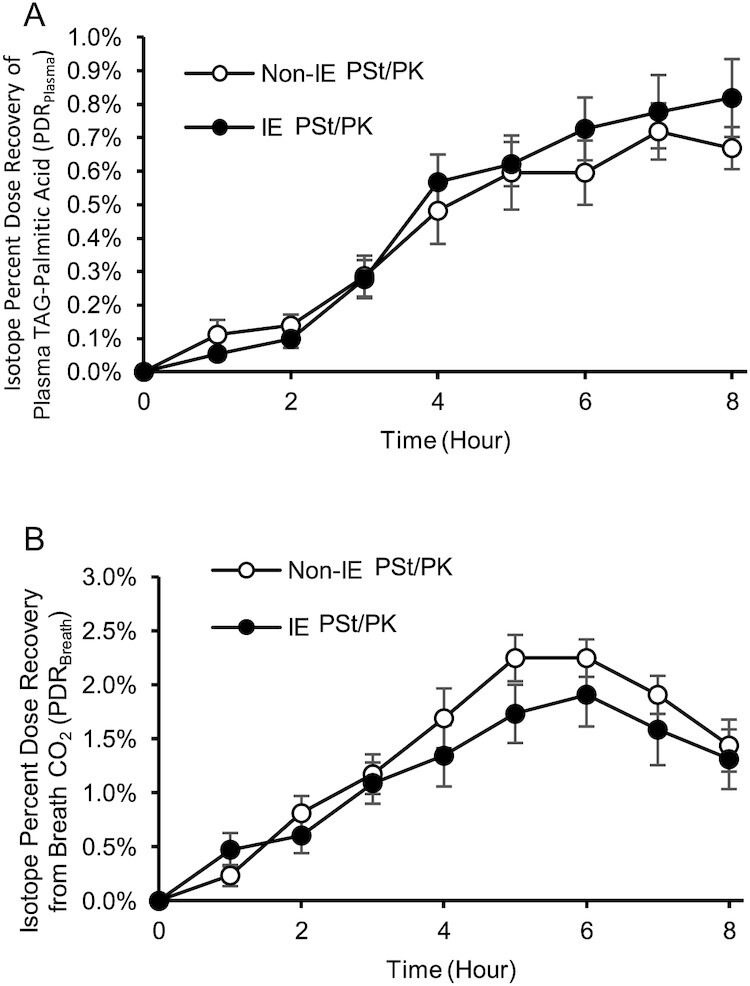
The effect of an interesterified (IE) palm stearin/palm kernel (PSt/PK) fat on postprandial [^13^C]palmitic acid recovery in (A) plasma triacyglycerol (TAG) palmitic acid and (B) CO_2_ from breath. Data are expressed as mean (SEM), *n* = 9, hourly percent dose recovery (PDR) of the total [^13^C] palmitic acid consumed as 75 mg [1,1,1]-^13^C glyceryl tripalmitin blended in the test fats used in the study meals. No differences were observed in (A) PDR_Plasma_ [incremental area under the curve (iAUC)] between IE and non-IE fats over the postprandial period. Non-IE (B) PDR_Breath_ (iAUC) was higher than IE over the postprandial period (*P* = 0.017). Data were analyzed using a linear mixed model.

No treatment differences were observed in the PDR_plasma_ for [^13^C]palmitic acid (iAUC) when comparing IE and non-IE fats (treatment effect *P* = 0.155; mean difference IE – non-IE: 0.019%; 95% CI: –0.007, 0.045) (**Figure 2**A). There was a significant main effect of meal for labeled palmitic acid oxidation as measured by PDR_Breath_ CO_2_ (iAUC), with a reduction in the appearance of breath ^13^C following IE fat compared with non-IE fat (Figure 2B: treatment effect *P* = 0.017; mean difference IE – non-IE: –0.19%; 95% CI: –0.34, –0.04).

### Postprandial lipoprotein particles

Postprandial lipoprotein subfraction particle concentrations and size are presented in **Table 4**. For comparisons between IE and non-IE fats, extra extra large (XXL)–VLDL/chylomicron (with average diameter >75 nm) particle concentrations were significantly higher following IE compared with non-IE (post hoc pairwise treatment effect *P =* 0.023), but there were no Bonferroni-adjusted time point–specific significant differences between the 2 fats. Otherwise, no differences between IE and non-IE were observed.

**TABLE 4:** Postprandial lipoprotein subclass particle concentrations and lipoprotein size following an interesterified (IE) palm stearin/palm kernel (PSt/PK) fat compared with a non-IE equivalent, relative to a reference rapeseed oil (RO)^1^

	Non-IE PSt/PK fat						IE PSt/PK fat						RO (reference)						Treatment	Time	Treatment × time
Characteristic	0 h	2 h	4 h	5 h	6 h	8 h	0 h	2 h	4 h	5 h	6 h	8 h	0 h	2 h	4 h	5 h	6 h	8 h	P value		
L-LDL-P concentration, nmol/L	156.6 (144.2, 169.0)	153.9 (148.5, 159.3)	145.1 (140.0, 150.2)	144.8 (140.3, 149.2)	144.9 (140.6, 149.3)	154.8 (149.4, 160.2)	151.5 (136.9, 166.1)	151.2 (147.2, 155.3)	144.0 (138.0, 149.9)	142.7 (136.7, 148.7)	142.7 (138.0, 147.4)	148.2 (126.4, 170.1)	145,9 (132.1, 157.6)	151.4 (147.5, 155.2)	145.2 (141.1, 149.3)	145.8 (139.8, 151.8)	147.5 (143.2, 151.8)	151.4 (144.4, 158.4)	NS	<0.001	NS
S-LDL-P concentration, nmol/L	146.0 (133.4, 158.5)	146.2 (140.6, 151.9)	137.7 (132.7, 142.6)	136.2 (131.9, 140.60)	133.2 (128.5, 137.9)	140.7 (135.1, 146.23)	145.0 (131.3, 158.7)	144.9 (140.2, 149.5)	135.4 (130.2, 140.5)	133.0 (127.5, 138.5)	131.6 (126.8, 136.4)	141.3 (129.3, 153.3)	133.4 (123.0, 143.9)	142.9 (138.5, 147.2)	137.8 (132.9, 142.6)	137.7 (131.6, 143.8)	136.1 (131.5, 140.7)	140.4 (134.0, 146.9)	NS	<0.001	NS
LDL-P size, nm	23.55 (23.54, 23.56)	23.52 (23.50, 23.53)	23.54 (23.52, 23.60)	23.55 (23.53, 23.57)	23.59 (23.57, 23.62)	23.62 (23.60, 23.65)	23.55 (23.53, 23.56)	23.51 (23.49, 23.53)	23.55 (23.53, 23.56)	23.56 (23.55, 23.58)	23.60 (23.58, 23.62)	23.63 (23.60, 23.66)	23.60 (23.59, 23.61)	23.54 (24.36, 23.56)	23.57 (23.55, 23.59)	23.59 (23.57, 23.61)	23.61 (23.59, 23.63)	23.61 (23.58, 23.63)	0.005	<0.001	NS
XL-HDL-P concentration, µmol/L	0.45 (0.36, 0.54)	0.42 (0.40, 0.43)	0.46 (0.44, 0.48)	0.48 (0.45, 0.50)	0.47 (0.45, 0.50)	0.50 (0.48, 0.52)	0.45 (0.36, 0.54)	0.43 (0.41, 0.45)	0.47 (0.45, 0.49)	0.49 (0.47, 0.52)	0.50 (0.47, 0.52)	0.52 (0.48, 0.56)	0.47 (0.39, 0.54)	0.43 (0.42, 0.45)	0.47 (0.45, 0.49)	0.49 (0.46, 0.51)	0.47 (0.44, 0.51)	0.48 (0.45, 0.51)	NS	<0.001	NS
L-HDL-P concentration, µmol/L	1.02 (0.79, 1.25)	1.00 (0.86, 1.13)	1.01 (0.88, 1.14)	1.02 (0.88, 1.16)	1.09 (0.95, 1.22)	1.24 (1.10, 1.39)	1.10 (0.87, 1.32)	0.99 (0.86, 1.12)	0.95 (0.82, 1.09)	0.97 (0.83, 1.11)	1.03 (0.90, 1.16)	1.20 (1.03, 1.37)	1.07 (0.88, 1.27)	0.99 (0.86, 1.12)	1.04 (0.91, 1.12)	1.08 (0.94, 1.22)	1.12 (0.98, 1.25)	1.18 (0.86, 1.13)	NS	<0.001	NS
M-HDL-P concentration, µmol/L	1.84 (1.73, 1.95)	1.74 (1.68, 1.80)	1.74 (1.68, 1.80)	1.75 (1.69, 1.80)	1.85 (1.80, 1.91)	2.04 (1.97, 2.11)	1.85 (1.72, 1.98)	1.74 (1.69, 1.80)	1.70 (1.65, 1.76)	1.73 (1.66, 1.80)	1.82 (1.77, 1.87)	2.03 (1.93, 2.14)	1.79 (1.69, 1.89)	1.75 (1.70, 1.80)	1.81 (1.74, 1.88)	1.84 (1.77, 1.91)	1.88 (1.81, 1.96)	1.95 (1.87, 2.03)	NS	<0.001	NS
S-HDL-P concentration, µmol/L	4.66 (4.52, 4.81)	4.53 (4.42, 4.64)	4.46 (4.35, 4.56)	4.42 (4.33, 4.52)	4.53 (4.42, 4.63)	4.68 (4.56, 4.80)	4.67 (4.49, 4.84)	4.51 (4.41, 4.62)	4.39 (4.30, 4.47)	4.36 (4.26, 4.47)	4.45 (4.36, 4.54)	4.66 (4.53, 4.80)	4.53 (4.39, 4.67)	4.47 (4.38, 4.56)	4.53 (4.38, 4.67)	4.53 (4.40, 4.67)	4.56 (4.42, 4.70)	4.61 (4.44, 4.79)	NS	<0.001	NS
HDL-P size, nm	9.95 (9.84, 10.07)	9.92 (9.90, 9.94)	9.95 (9.93, 9.97)	9.97 (9.94, 10.01)	9.98 (9.95, 10.02)	10.04 (10.01, 10.07)	9.95 (9.82, 10.07)	9.93 (9.91, 9.95)	9.96 (9.93, 9.99)	9.99 (9.96, 10.01)	10.01 (9.98, 10.03)	10.05 (9.99, 10.11)	9.98 (9.87, 10.09)	9.94 (9.92, 9.96)	9.97 (9.95, 10.00)	10.00 (9.97, 10.02)	10.00 (9.97, 10.02)	10.02 (9.98, 10.05)	NS	<0.001	NS
XXL-VLDL/chylomicron–P concentration, nmol/L	0.13 (0.10, 0.16)	0.16 (0.14, 0.19)^3^	0.27 (0.24, 0.31)	0.31 (0.27, 0.35)	0.27 (0.24, 0.31)	0.22 (0.19, 0.25)^2^	0.14 (0.10, 0.19)	0.17 (0.14, 0.20)	0.31 (0.27, 0.35)	0.35 (0.28, 0.41)	0.32 (0.26, 0.38)^2^	0.25 (0.21, 0.29)^3^	0.13 (0.09, 0.17)	0.20 (0.17, 0.22)	0.26 (0.23, 0.29)	0.26 (0.23, 0.30)	0.21 (0.17, 0.25)	0.16 (0.11, 0.21)	<0.001	<0.001	<0.001
XL-VLDL-P concentrations, nmol/L	0.58 (0.39, 0.77	0.74 (0.63, 0.84)^3^	1.18 (1.07, 1.29)	1.38 (1.26, 1.51)	1.26 (1.11, 1.41)^2^	0.99 (0.87, 1.11)^2^	0.63 (0.39, 0.60)	0.74 (0.65, 0.83)^3^	1.24 (1.1, 1.4)	1.41 (1.20, 1.63)	1.37 (1.12, 1.61)	1.16 (0.96, 1.36)^3^	0.55 (0.34, 0.76)	0.88 (0.79, 0.91)	1.16 (1.03, 1.29)	1.16 (1.01, 1.31)	0.93 (0.77, 1.09)	0.67 (0.45, 0.90)	<0.001	<0.001	0.004
L-VLDL-P concentrations, nmol/L	4.10 (2.99, 5.21)	4.70 (4.20, 5.20)^3^	6.59 (6.13, 7.04)	7.57 (7.05, 8.10)	7.12 (6.43, 7.80)^2^	5.74 (5.16, 6.33)	4.59 (3.17, 6.00)	4.70 (4.21, 5.18)^2^	6.91 (6.27, 7.55)	7.66 (6.89, 8.43)	7.57 (6.58, 8.56)^2^	6.39 (5.35, 7.43)^3^	3.71 (2.60, 4.83)	5.47 (5.03, 5.90)	6.73 (6.16, 7.31)	6.68 (6.02, 7.33)	5.65 (4.91, 6.38)	4.46 (3.37, 5.56)	<0.001	<0.001	<0.001
M-VLDL-P concentrations, nmol/L	15.61 (12.59, 18.63)	17.10 (15.88, 18.32)	20.83 (19.74, 21.91)	22.93 (21.80, 24.05)	21.74 (20.20, 23.29)	18.82 (17.32, 20.32)	16.35 (12.57, 20.12)	16.65 (15.28, 18.02)	20.83 (19.22, 22.46)	22.54 (20.85, 24.22)	22.29 , (20.34, 24.24)	20.02 (17.26, 22.7)	14.23 (11.49, 17.00)	18.98 (17.81, 20.15)	21.88 (20.60, 23.17)	21.61 (20.03, 23.18)	19.38 (17.45, 21.31)	16.31 (13.44, 19.18)	NS	<0.001	0.001
S-VLDL-P concentrations, nmol/L	27.84 (24.28, 31.40)	28.71 (27.50, 29.93)	30.47 (29.32, 31.62)	31.87 (30.76, (32.99)	30.83 (29.31, 32.34)	29.25 (27.92, 30.58)	28.49 (24.01, 32.97)	28.60 (27.13, 30.08)	30.79 (29.60, 31.99)	31.48 (30.41, 32.55)	30.91 (29.64, 32.18)	30.15 (27.25, 33.05)	25.99 (22.88, 29.10)	30.09 (28.95, 31.25)	32.66 (31.42, 33.90)	32.59 (30.98, 34.20)	30.71 (29.06, 32.36)	27.91 (24.90, 30.92)	NS	<0.001	NS
VLDL-P size, nm	36.70 (36.18, 37.23)	37.05 (36.84, 37.26)^2^	37.89 (37.68, 38.10)	38.21 (38.01, 38.41)	37.95 (37.72, 38.18)^3^	37.41 (37.14, 37.67)^2^	36.76 (36.13, 37.38)	37.03 (36.82, 37.25)^2^	37.92 (37.66, 38.18)	38.14 (37.83, 38.45)	37.99 (37.63, 38.34)^2^	37.61 (37.14, 38.08)^3^	36.60 (36.09, 37.10)	37.32 (37.13, 37.52)	37.84 (37.60, 38.08)	37.73 (37.40, 38.05)	37.29 (36.92, 37.66)	36.64 (36.10, 37.19)	<0.001	<0.001	<0.001

^1^Estimated marginal means (95% CI) at 2, 4, 5, 6, and 8 h, *n* = 20. Significant differences between treatments at individual time points are indicated by superscript numbers (data analyzed using a linear mixed model with postprandial response as dependent variable; treatment, time, period, treatment × time, and treatment × period as fixed effects; baseline values as covariate; participant as random effect; and post hoc pairwise comparisons adjusted for multiple comparisons using Bonferroni). Significantly different from RO. There were no significant differences at individual time points between IE and non-IE PSt/PK fats. There were no sex effects or sex × treatment interactions. There were treatment × period interactions for L-HDL-P (*P* < 0.01), M-HDL-P (*P* < 0.05), HDL size (*P* < 0.05), and S-VLDL-P (*P* < 0.05). L, large; M, medium; P, particle; S, small; XL, extra large; XXL, extra extra large.

2 *P* < 0.05.

3 *P* < 0.01.

There were a number of differences between IE/non-IE fats and RO. LDL size was significantly lower following IE and non-IE fats relative to RO (*P* = 0.005). VLDL size was reduced by palmitic acid–rich fats at 2 h relative to RO (IE, *P* = 0.008; non-IE, *P* = 0.013) and increased in the late postprandial period compared with RO (6 h IE, *P* = 0.054; 6 h non-IE, *P* = 0.012; 8 h IE, *P* = 0.007; 8 h non-IE, *P* = 0.026). There were significantly lower (2 h) and higher (6–8 h) concentrations of XXL-VLDL, extra large (XL)–VLDL, and large-VLDL particles following both IE and non-IE fats compared with RO (Table 4), whereas there were no treatment effects for medium-VLDL and small-VLDL particle concentrations. Analysis by sex did not reveal any significant differences in postprandial lipoprotein particle concentrations or particle sizes between men and women or treatment × sex interactions.

### Chylomicron fraction apoB48 concentrations

**FIGURE 3:**
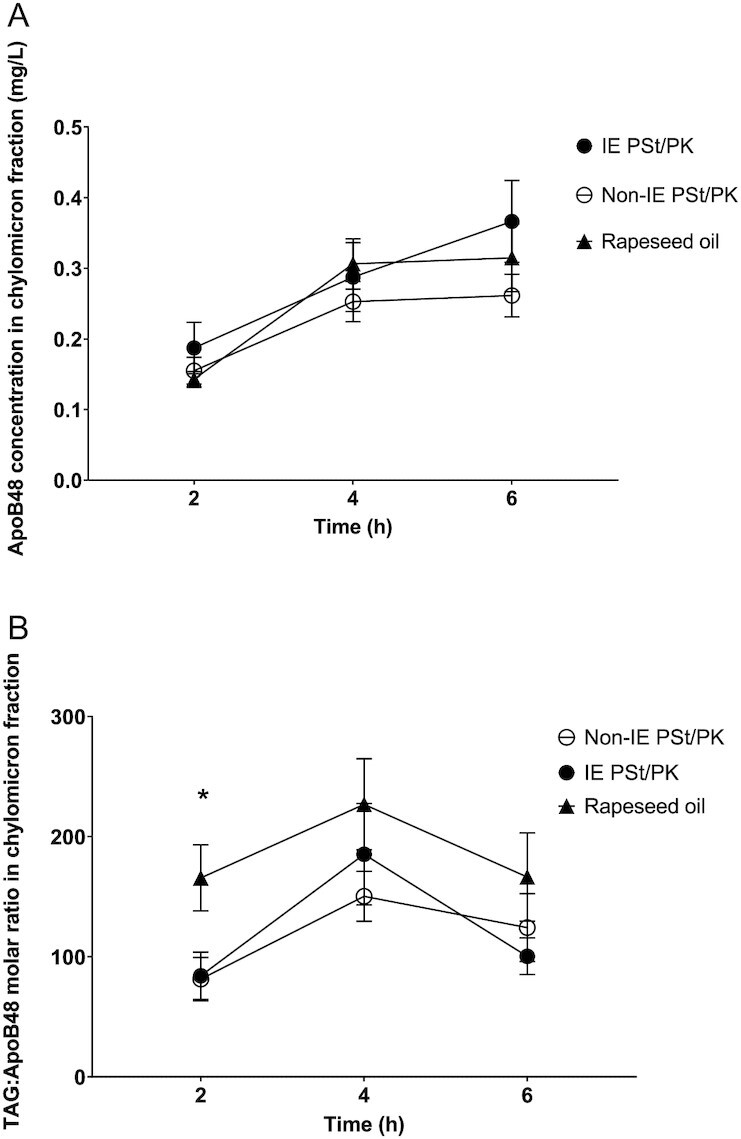
The effect of an interesterified (IE) palm stearin/palm kernel (PSt/PK) fat on postprandial (A) apolipoprotein B48 (apoB48) concentrations and (B) triacyglycerol (TAG)/apoB48 molar ratio relative to a non-IE equivalent and a reference rapeseed oil (RO). Data are mean (SEM), *n* = 20. Data were analyzed using a linear mixed model (dependent variable natural log of triacylglycerol; LnTAG /apoB48 ratio; fixed factors of treatment, time, period, treatment × period, treatment × time; and participant as a random effect). Post hoc analysis of time point differences with Bonferroni adjustment showed significant differences between both IE and non-IE fats compared with RO at 2 h: *IE compared with RO *P* = 0.002, non-IE compared with RO *P* = 0.004.

There were no effects of meal type on chylomicron fraction apoB48 concentrations (**Figure 3**A). There were no differences in postprandial TAG/apoB48 molar ratio (chylomicron size) between IE and non-IE fats, but ratios of TAG molecules to apoB48 molecules were significantly lower following both IE (*P* = 0.00006) and non-IE (*P* = 0.00002) fats compared with RO, with post hoc time point analysis showing differences at 2 h only (Figure 3B). This indicates larger chylomicrons following RO compared with the palm-based fats in the early postprandial period. There were no sex effects or treatment × sex interactions.

### Plasma triacylglycerol fatty acid composition

There was a very small but significant difference between IE and non-IE fats for plasma TAG fraction palmitic acid (16:0), with higher concentrations of plasma 16:0 following IE compared with the non-IE equivalent (treatment effect *P* = 0.011), with no treatment × time interaction (**Supplementary Figure 3A**). Post hoc comparisons at individual time points showed that pairwise differences were only statistically significant at 8 h (mean difference IE – non-IE: 0.66 mg/L; 95% CI: 0.15, 1.19; *P* = 0.011). As expected, there were significant differences between both fats compared with RO for 16:0 (treatment effect *P* < 0.001), 16:1n–9 (*P* < 0.001, data not shown), 18:0 (*P* < 0.001), 18:1n–9 (*P* < 0.001), and 18:2 (*P* < 0.001) (Supplementary Figure 3A–D).

### In vitro analysis: Rate of delivery and free fatty acids production during simulated gastric digestion

**FIGURE 4:**
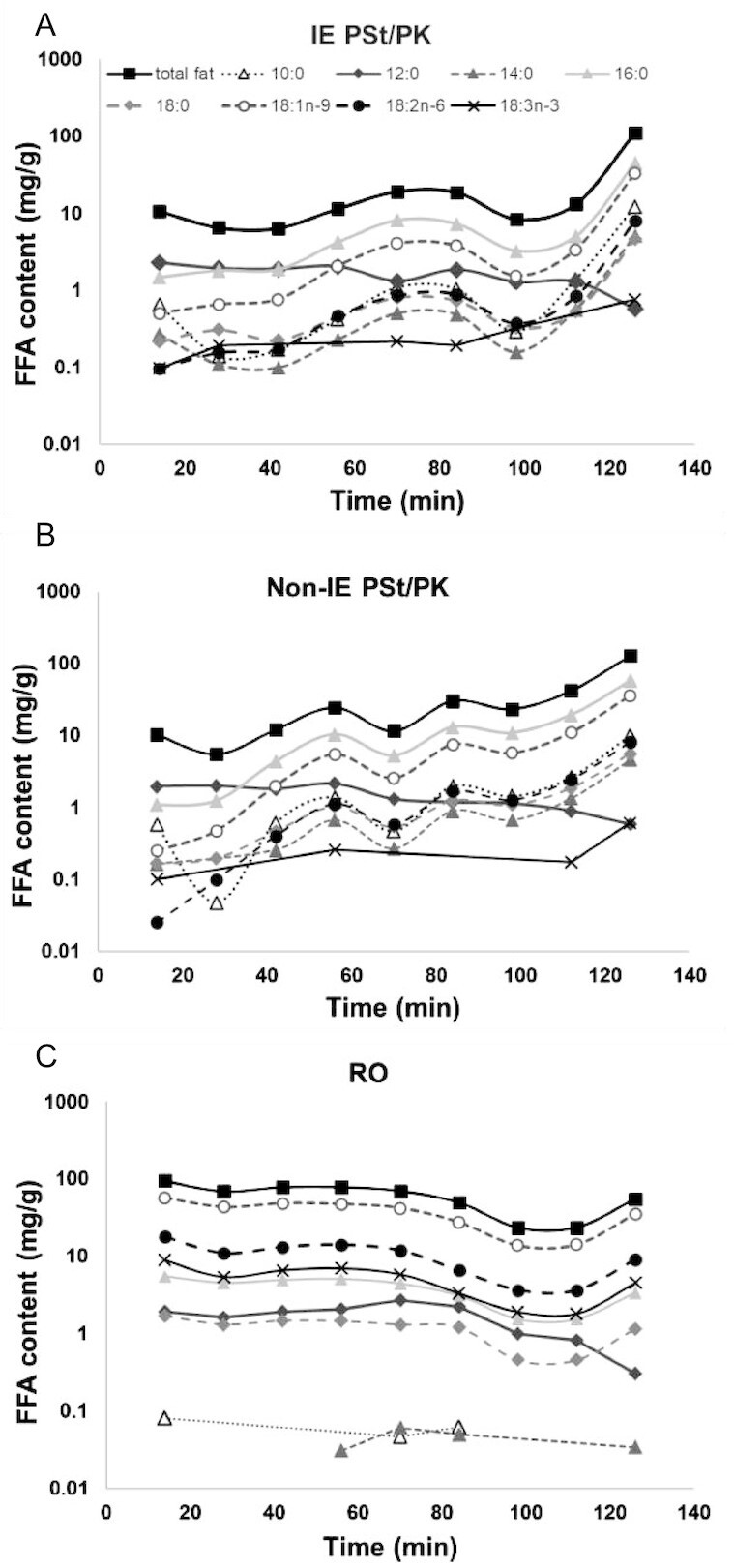
Free fatty acid and total fat concentrations of samples emptied from the dynamic gastric model during simulated gastric digestion at specific time points (14 to 126 min) for muffins made from (A) interesterified palm stearin/palm kernel (IE PSt/PK), (B) non-IE PSt/PK, and (C) rapeseed oil (RO).

There was clear phase separation observed in gastric samples of IE and non-IE (see **Supplementary Figure 4**). The profile of free fatty acids (FFAs) released at each time point during gastric digestion is shown in **Figure 4**. The trend followed the overall fat composition of IE, non-IE, and RO, except for lauric acid (C12), in which a more consistent rate of emptying was detected, with a slight dilution over time. Since lauric acid is not a RO fatty acid, and the concentrations are consistent between all 3 samples, it is likely that it has come from the milkshake component, probably from the lecithin used to stabilize the milkshake powder. For IE and non-IE fats, the total FFA content emptied from the DGM was very low in the early time points (∼10 mg/g, compared with ∼100 mg/g for RO) and then increased toward the final stages, consistent with the observed creaming effect (Supplementary Figure 4) delaying the emptying of the fat from the DGM. For the RO sample, the FFA concentrations were more consistent, gradually decreasing across the time points. For IE and non-IE, C12 was the most abundant FFA emptied in the first 2 time points, then dropping down to the least at the end. In RO, the lauric acid profile was more similar to the other fats. A linear relation was found plotting the FFA profiles against the total fat emptied (apart from C12), suggesting that each FFA released followed the trend of the total fat (results not shown).

## Discussion

In light of the widespread use of IE fats, the aim of the current study was to investigate the impact of a widely used IE fat on postprandial metabolism compared with its non-IE counterpart and a high MUFA control oil. Contrary to our hypothesis, we did not observe an effect of interesterification on postprandial lipemia. However, a novel finding was the unfavorable pattern of late postprandial lipoprotein particles elicited following the SFA-rich fats (IE and non-IE fats) compared with the MUFA-rich oil (RO).

In contrast to our current findings, we previously reported a greater postprandial lipemia following the interesterification of PSt/PK (80:20) compared with its non-IE counterpart in the first 0–4 h (12). We had proposed that the higher postprandial response following the IE fat, in the absence of differences in solid fat at body temperature (both between 22% and 25%), was due to the higher proportion of palmitic acid in the *sn*-2 position of the IE fat (53 mol%) compared with the non-IE fat (33 mol%), as previously reported in animal and human infant studies (15, 27). In partial support of the theory that digestion and absorption of SFAs are superior when present in the *sn*-2 position, we observed a slightly greater palmitic acid concentration in the plasma TAG fraction following IE fat (higher *sn*-2 SFA) compared with non-IE fat (lower *sn*-2 SFA) (Supplementary Figure 3A). However, this small difference in plasma TAG fraction concentrations did not extend to differences in lipemia as measured by total plasma TAG, the appearance of [^13^C]palmitic acid in plasma, or gastric emptying rates measured in vitro. A possible explanation for in vivo differences between studies is the differences in characteristics of the study populations. In the current study, the average age was 58 y compared with 20 y in the previous study using the same test meal challenge (nutrient composition) and same test fats (12). Postprandial lipemia increases with age (28) and metabolic flexibility reduces with age (e.g., reduced insulin sensitivity) (29). Therefore, the lack of difference between IE and non-IE fats in the current study may be due to a less “efficient” metabolic response, masking any subtle differences in metabolism elicited by differences in the *sn*-2 positional composition of the test fats.

Interestingly, both palmitic acid–rich fats resulted in a lower lipemia compared with the high-MUFA oil, in agreement with our previous studies (10, 11). This may be due to the difference in solid fat content at body temperature between fats (∼25% difference), in agreement with previous work showing that solid fat attenuates the postprandial lipemic response (11). Alternatively, it may be a consequence of the larger chylomicrons produced following MUFA-rich oils compared with SFA-rich fats, which are cleared from the circulation more rapidly than smaller chylomicron particles (30) and/or differential effects of SFA- compared with MUFA-rich fats on gastric emptying (31). Indeed, in the current study, the palmitic acid–rich fats resulted in smaller chylomicron particles postprandially compared with the MUFA-rich oil, as measured by the lower TAG/apoB48 ratio. In addition, in line with the more pronounced attenuation in lipemia in the early phase (0–4 h), chylomicron particle concentrations (the XXL-VLDL particles measured by NMR; >75 nm) were lower during the early postprandial phase (2 h), and both XXL-VLDL particles and VLDL particle size were higher during the late phase (6–8 h) following the palmitic acid–rich fats compared with the MUFA-rich oil, indicating a more rapid removal of MUFA-containing chylomicrons. Furthermore, in vitro gastric digestion demonstrated phase separation for the palmitic acid–rich fat muffins, which is likely to be due to their high solid fat content, resulting in a change in buoyancy and delayed gastric emptying of the fats that had creamed to the top of the digesta. Indeed, the work by Thilakarathna et al. (32) demonstrates that there is a complex interplay between the physical state of dietary fat and its colloidal properties, which impact digestion and lipolysis. Taken together with the differences in lipoproteins, this suggests that both delayed gastric emptying of the higher melting point SFA-rich fats and more rapid removal of the MUFA-rich oil may account for the observed differences in postprandial lipemia.

A novel finding of the current study was the unfavorable lipoprotein profile, despite the lower lipemic response following the palmitic acid–rich fats compared with the MUFA-rich oil, namely, smaller LDL particle size and higher XL-VLDL particle concentrations at 6–8 h. XL-VLDL has a mean size of 64 nm and, unlike the larger XXL-VLDL (representative of chylomicrons postprandially), is thus reflective of TAG-rich lipoprotein (TRL; chylomicrons and VLDL) remnant particles (33), which are associated with an increased risk of CVD (34, 35). The importance of postprandial lipemia in relation to CVD risk is gaining recognition (36), and large epidemiologic studies have shown that elevated nonfasting (postprandial) TAG is an independent risk factor for CVD in part mediated by the generation of atherogenic lipoproteins in the postprandial phase [reviewed elsewhere (7)]. During the postprandial phase, lipoprotein remodeling occurs, mediated by cholesteryl ester transfer protein, resulting in the reciprocal exchange of TAG and cholesterol between TRL and HDL and LDL. If postprandial lipemia is sustained over a prolonged period of time, this reciprocal exchange is enhanced, resulting in TAG-enriched LDL and HDL particles and cholesterol-enriched remnant TRL, which are atherogenic (37). Furthermore, studies have found that the concentration of lipemia is associated with the extent of this lipoprotein remodeling (38). The more favorable lipoprotein profile observed after the MUFA-rich oil, despite the higher concentration of lipemia, may explain the association of dietary MUFA with reduced CVD risk and inform recent debates regarding the relevance of postprandial lipemia and CVD risk (39). The adverse lipoprotein profile observed following the palmitic acid–rich fats therefore adds to the body of mechanistic evidence relating to the adverse effect of SFA-rich fats on health (40) and highlights the importance of looking beyond the traditionally measured postprandial TAG features when exploring postprandial lipid associations with disease (41).

Although the lipemic responses between the IE and non-IE fats did not differ, there were small differences in lipoprotein particle numbers and breath ^13^C between the IE and non-IE fats, namely, higher XXL-VLDL particles and lower breath ^13^C in the postprandial period following IE consumption. Further studies are required to understand the XXL-VLDL particle concentration findings to rule out potential type I errors (42). The difference in breath ^13^C was an unexpected finding, suggesting a difference in the availability of palmitic acid for oxidation between the IE and non-IE meals. However, the ^13^C label was incorporated into the fat as tripalmitin; palmitic acid intrinsic to the IE and non-IE TAG was not labeled. Without an understanding of the fate of the tracer at the TAG resynthesis/chylomicron assembly stage in the enterocyte, any interpretation of these effects on the appearance of ^13^C in breath can only be speculative.

There were significant treatment × period interactions for the change from baseline in plasma TAG and also for large-HDL-P (particles), medium-HDL-P, and small-VLDL-P concentrations and HDL size. However, there were no treatment × period interactions for the primary outcome (8-h iAUC for plasma TAG) or other summary measures of postprandial lipemia, suggesting that this did not represent a carryover effect. Furthermore, the possibility of a 7-d carryover effect is implausible for a single high-fat meal in which the outcomes of interest are only expected to change during the postprandial period.

## Conclusion

In conclusion, the process of interesterification did not modify postprandial lipemia. However, the palmitic acid–rich fats, irrespective of interesterification, resulted in an attenuated lipemic response compared with a MUFA-rich oil, which, in part, may be due to delayed gastric emptying or altered chylomicron size and composition. The greater preponderance of proatherogenic large TRL remnant particles and small LDL particles in the late postprandial phase following the palmitic acid–rich fats relative to the MUFA-rich oil adds a new postprandial dimension to the body of mechanistic evidence linking SFA to increased risk of CVD and demonstrates the importance of looking beyond TAG when exploring postprandial lipid-mediated associations with CVD.

## Supplementary Material

nqaa413_Supplemental_File

## Data Availability

Data described in the manuscript, code book, and analytic code will be made available upon request pending application and approval.

## Notes

This work was funded by a Biotechnology and Biological Sciences Research Council–Diet and Health Research Industry Club (BBSRC-DRINC) grant (BB/N020987/1) and BBSRC equipment grant (BB/R00028X/). Test fats for the dietary intervention were supplied by ADM Speciality Oils & Fats Ltd.

WLH and SEB are joint senior authors.

Supplemental Figures 1–3 are available from the “Supplementary data” link in the online posting of the article and from the same link in the online table of contents at https://academic.oup.com/ajcn/.

Abbreviations used: APE, atom % excess; apoB48, apolipoprotein B48; CVD, cardiovascular disease; DGM, dynamic gastric model; FAME, fatty acid methyl ester; FFA, free fatty acid; GC-IRMS, gas chromatography–isotope ratio mass spectrometry; iAUC, incremental area under the curve; IE, interesterified; MRU, Metabolic Research Unit; NEFA, nonesterified fatty acid; PDR, percent dose recovery; PK, palm kernel; PSt, palm stearin; RO, rapeseed oil; SSF, simulated salivary fluid; TAG, triacylglycerol; TLC, thin-layer chromatography; TRL, triglyceride-rich lipoprotein; VPDB, Vienna Pee Dee Belemnite; XL, extra large; XXL, extra extra large.
