# Supplementary material for: Palmitic acid–rich oils with and without interesterification lower postprandial lipemia and increase atherogenic lipoproteins compared with a MUFA-rich oil: A randomized controlled trial
Source: Am J Clin Nutr. 2021 Mar 1;113(5):1221–31. doi: 10.1093/ajcn/nqaa413 (PMC8106759; doi:10.1093/ajcn/nqaa413)
Supplement: nqaa413_Supplemental_File [file nqaa413_supplemental_file.pptx]

## Slide 1
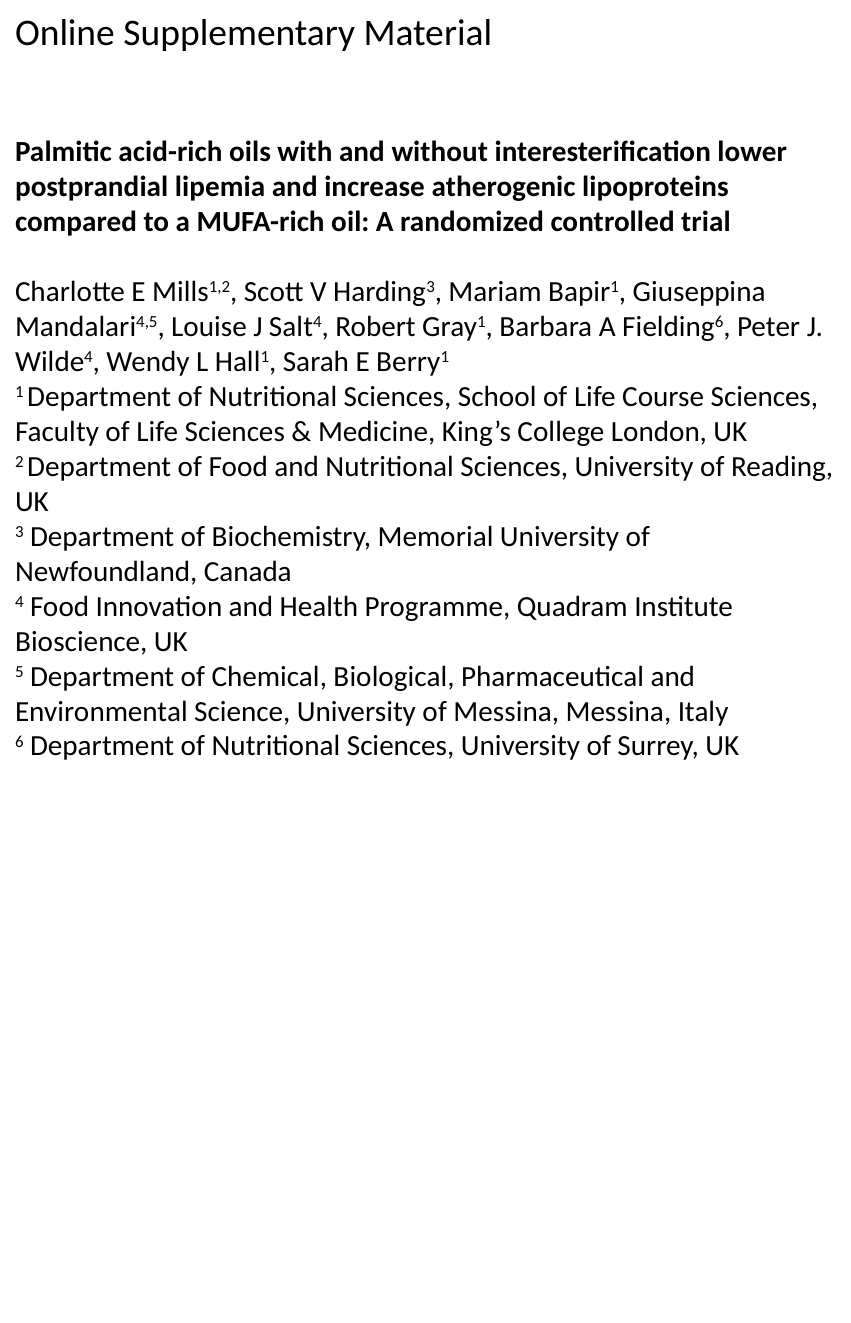

Online Supplementary Material
Palmitic acid-rich oils with and without interesterification lower postprandial lipemia and increase atherogenic lipoproteins compared to a MUFA-rich oil: A randomized controlled trial
Charlotte E Mills1,2, Scott V Harding3, Mariam Bapir1, Giuseppina Mandalari4,5, Louise J Salt4, Robert Gray1, Barbara A Fielding6, Peter J. Wilde4, Wendy L Hall1, Sarah E Berry1
1 Department of Nutritional Sciences, School of Life Course Sciences, Faculty of Life Sciences & Medicine, King’s College London, UK
2 Department of Food and Nutritional Sciences, University of Reading, UK
3 Department of Biochemistry, Memorial University of Newfoundland, Canada
4 Food Innovation and Health Programme, Quadram Institute Bioscience, UK
5 Department of Chemical, Biological, Pharmaceutical and Environmental Science, University of Messina, Messina, Italy
6 Department of Nutritional Sciences, University of Surrey, UK

## Slide 2
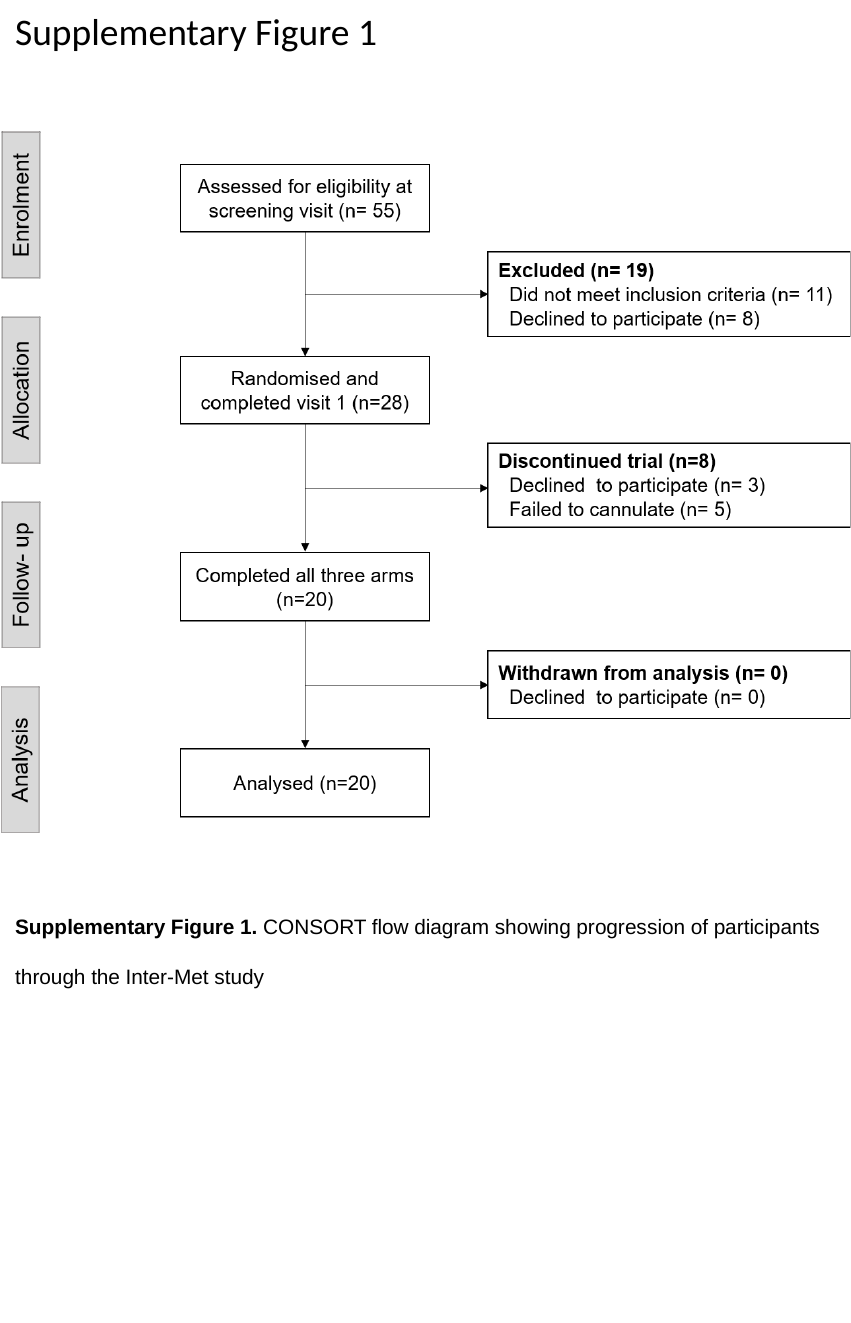

Supplementary Figure 1
Supplementary Figure 1. CONSORT flow diagram showing progression of participants through the Inter-Met study

## Slide 3
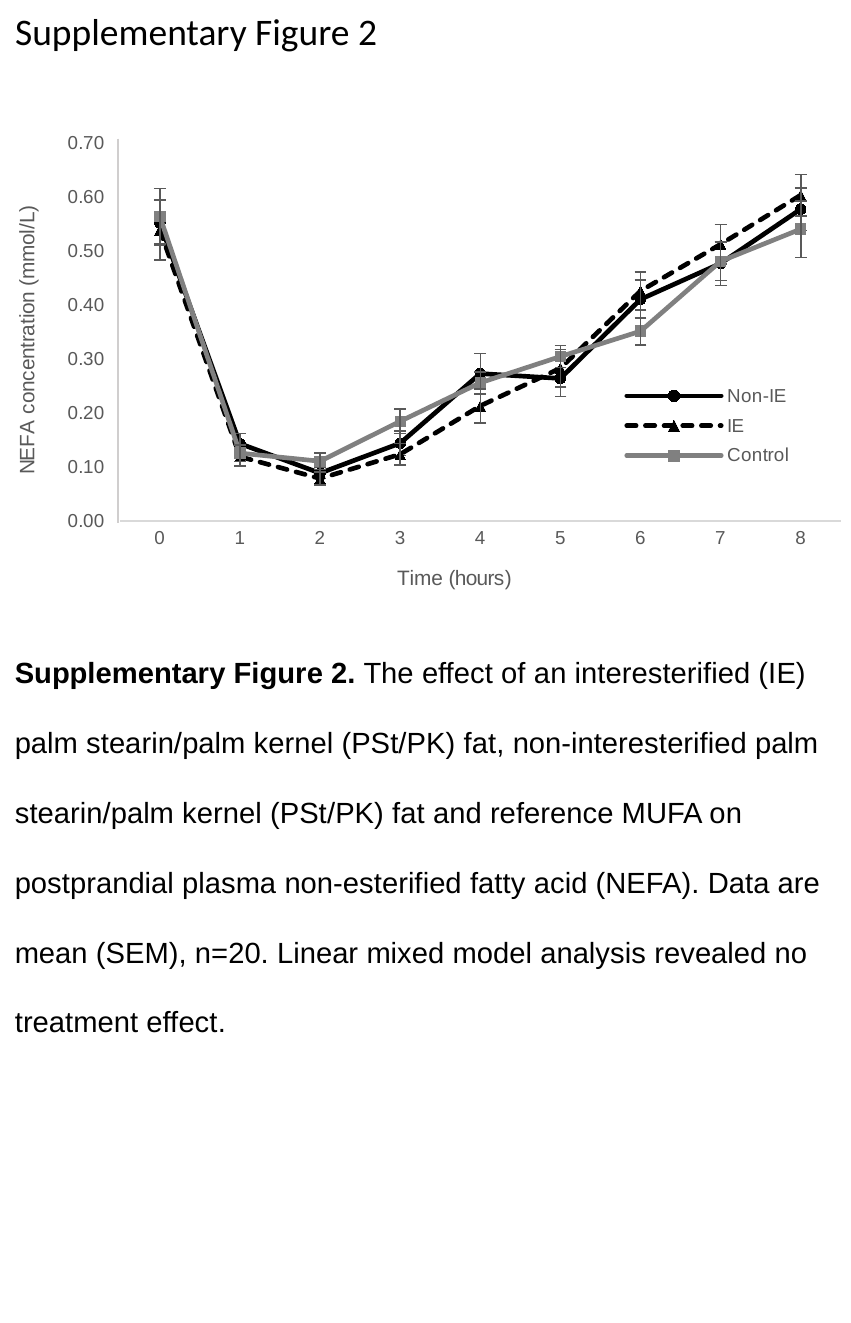

Supplementary Figure 2
### Chart
| Category | Non-IE | IE | Control |
|---|---|---|---|
| 0 | 0.5525 | 0.5395000000000001 | 0.5645 |
| 1 | 0.1435 | 0.11949999999999998 | 0.12599999999999997 |
| 2 | 0.08900000000000002 | 0.07900000000000001 | 0.11099999999999999 |
| 3 | 0.14450000000000002 | 0.12400000000000003 | 0.185 |
| 4 | 0.2735000000000001 | 0.21350000000000002 | 0.25650000000000006 |
| 5 | 0.2645 | 0.28350000000000003 | 0.30500000000000005 |
| 6 | 0.41100000000000003 | 0.4265 | 0.352 |
| 7 | 0.47750000000000015 | 0.5130000000000001 | 0.48150000000000015 |
| 8 | 0.5780000000000001 | 0.6039999999999999 | 0.541 |Supplementary Figure 2. The effect of an interesterified (IE) palm stearin/palm kernel (PSt/PK) fat, non-interesterified palm stearin/palm kernel (PSt/PK) fat and reference MUFA on postprandial plasma non-esterified fatty acid (NEFA). Data are mean (SEM), n=20. Linear mixed model analysis revealed no treatment effect.

## Slide 4
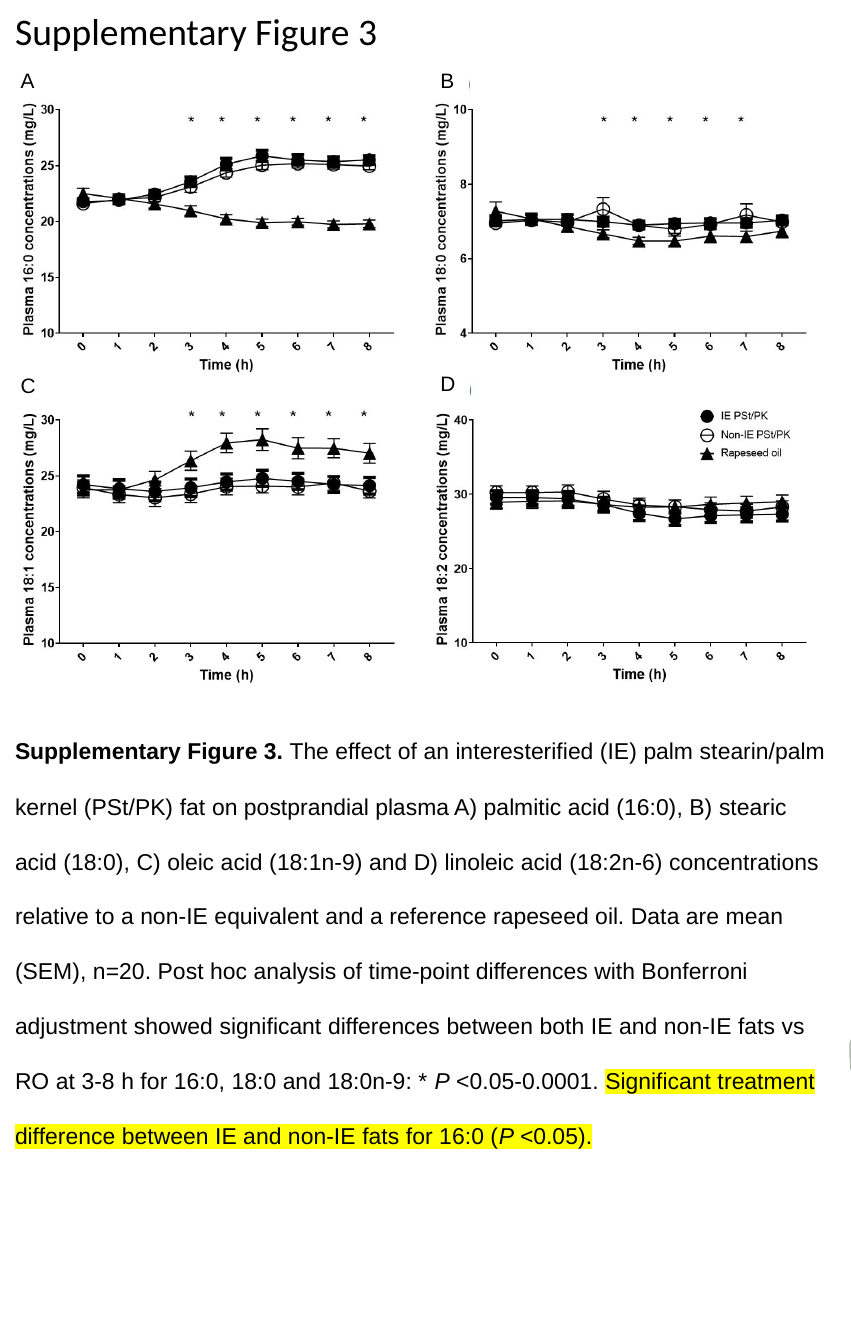

Supplementary Figure 3
A
B
D
C
Supplementary Figure 3. The effect of an interesterified (IE) palm stearin/palm kernel (PSt/PK) fat on postprandial plasma A) palmitic acid (16:0), B) stearic acid (18:0), C) oleic acid (18:1n-9) and D) linoleic acid (18:2n-6) concentrations relative to a non-IE equivalent and a reference rapeseed oil. Data are mean (SEM), n=20. Post hoc analysis of time-point differences with Bonferroni adjustment showed significant differences between both IE and non-IE fats vs RO at 3-8 h for 16:0, 18:0 and 18:0n-9: * P <0.05-0.0001. Significant treatment difference between IE and non-IE fats for 16:0 (P <0.05).

## Slide 5
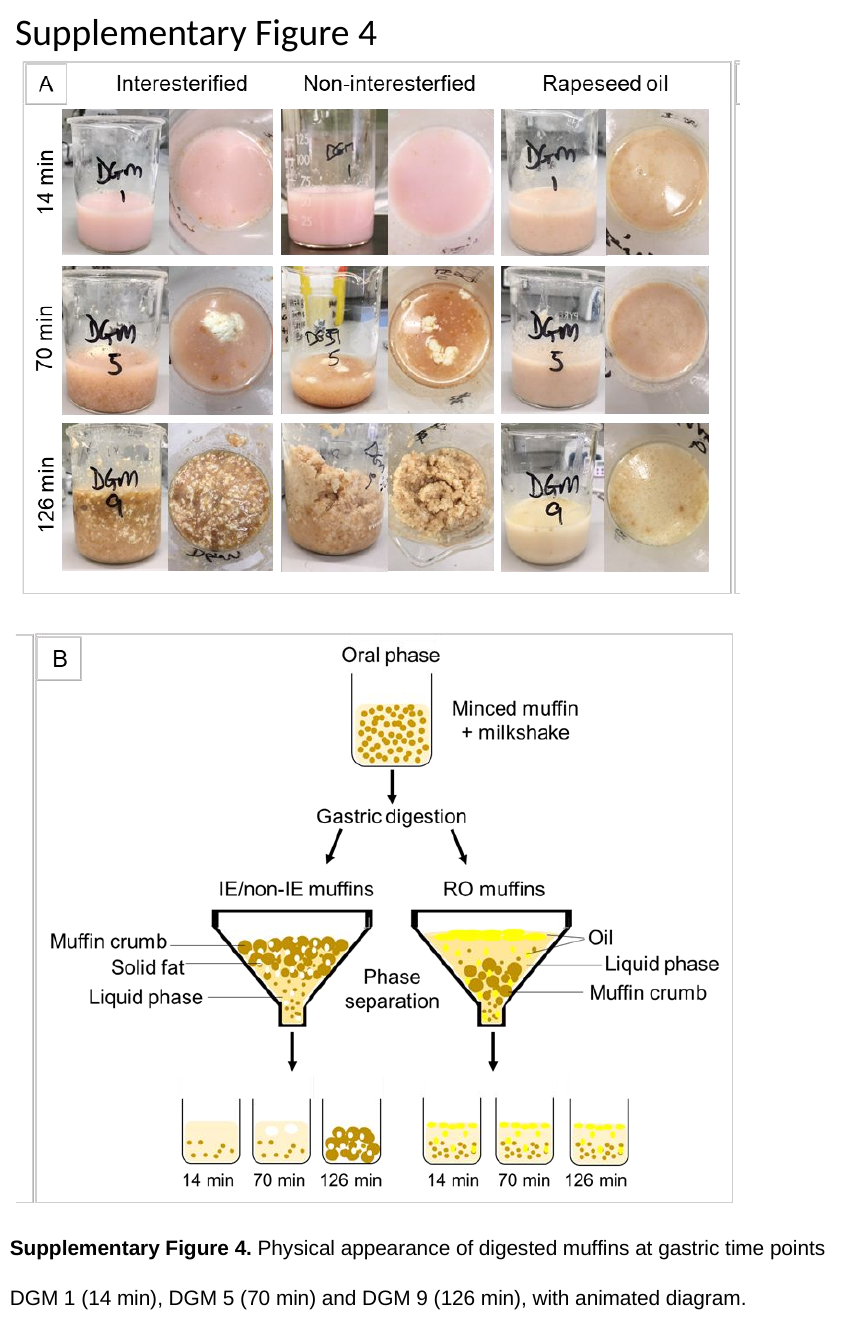

Supplementary Figure 4
Supplementary Figure 4. Physical appearance of digested muffins at gastric time points DGM 1 (14 min), DGM 5 (70 min) and DGM 9 (126 min), with animated diagram.
